# The volumetric-tumour histogram-based analysis of intravoxel incoherent motion and non-Gaussian diffusion MRI: association with prognostic factors in HER2-positive breast cancer

**DOI:** 10.1186/s12967-019-1911-6

**Published:** 2019-07-02

**Authors:** Chao You, Jianwei Li, Wenxiang Zhi, Yanqiong Chen, Wentao Yang, Yajia Gu, Weijun Peng

**Affiliations:** 1 Department of Radiology, Fudan University Cancer Center, Department of Oncology, Shanghai Medical College, Fudan University, No. 270, Dongan Road, Shanghai, 200032 People’s Republic of China; 2 Department of Breast Surgery, Key Laboratory of Breast Cancer in Shanghai, Fudan University Shanghai Cancer Center, Department of Oncology, Shanghai Medical College, Fudan University, Shanghai, People’s Republic of China; 3 Department of Ultrasound, Fudan University Cancer Center, Department of Oncology, Shanghai Medical College, Fudan University, Shanghai, People’s Republic of China; 4 Department of Pathology, Fudan University Shanghai Cancer Center, Department of Oncology, Shanghai Medical College, Fudan University Shanghai, Shanghai, People’s Republic of China

**Keywords:** Breast cancer, HER2-positive, Intravoxel incoherent motion, Non-Gaussian diffusion, Histogram-based analysis

## Abstract

**Background:**

To evaluate the imaging biomarkers of human epidermal growth factor receptor 2 (HER2) positive breast cancer in comparison to other molecular subtypes and to determine the feasibility of identifying hormone receptor (HR) status and lymph node metastasis status using volumetric-tumour histogram-based analysis through intravoxel incoherent motion (IVIM) and non-Gaussian diffusion.

**Methods:**

This study included 145 breast cancer patients with 148 lesions between January and November in 2018. Among the 148 lesions, 74 were confirmed to be HER2-positive. The volumetric-tumour histogram-based features were extracted from the combined IVIM and non-Gaussian diffusion model. IVIM and non-Gaussian diffusion parameters obtained from images of the subjects with different molecular prognostic biomarker statuses were compared by Student’s t test or the Mann–Whitney *U* test. The area under the curve (AUC), sensitivity, and specificity at the best cut-off point were reported. The Spearman correlation coefficient was calculated to analyse the correlations of clinical tumor nodule metastasis (TNM) stage and Ki67 with the IVIM and non-Gaussian diffusion parameters.

**Results:**

The entropy of mean kurtosis (MK) was significantly higher in the HER2-positive group than in the HER2-negative group (p = 0.015), with an AUC of 0.629 (95% CI 0.546, 0.707), a sensitivity of 62.6%, and a specificity of 66.2%. For HR status, the MD 5th percentile was higher in the HR-positive group of HER2-positive breast cancer (p = 0.041), with an AUC of 0.643 (95% CI 0.523, 0.751), while for lymph node status, the entropy of mean diffusivity (MK) was lower in the lymph node positive group (p = 0.040), with an AUC of 0.587 (95% CI 0.504, 0.668). The clinical TNM stage and Ki67 index were correlated with several histogram parameters.

**Conclusion:**

Volumetric-lesion histogram analysis of IVIM and the non-Gaussian diffusion model can be used to provide prognostic information about HER2-positive breast cancers and potentially contribute to individualized anti-HER2 targeted therapy plans .

**Electronic supplementary material:**

The online version of this article (10.1186/s12967-019-1911-6) contains supplementary material, which is available to authorized users.

## Background

Breast cancer is the most common malignancy in women and is considered potential heterogeneous [1]. Human epidermal growth factor receptor 2 (HER2) positivity, accounting for approximately 15–20% of breast cancers, is defined by HER2 protein overexpression measured by immunohistochemistry (IHC) status (IHC3+) or by fluorescence in situ hybridization (FISH) analysis. The success of targeted neoadjuvant therapy such as trastuzumab is especially accepted in HER2-positive breast cancer [2]. To date, on the basis of the negativity of hormone-receptor (HR) and positivity of lymph node status, dual anti-HER2 therapy of the combination of trastuzumab and pertuzumab was associated with an increased proportion of HER2-positive patients achieving a better prognosis [3, 4].

Breast magnetic resonance imaging (MRI) is useful for detecting breast cancer and guiding treatment plans [5, 6]. Diffusion-weighted imaging (DWI) can differentiate benign and malignant breast lesion, to predict the response to neoadjuvant chemotherapy (NAC) and determine associated prognostic factors [7, 8]. Because the putative apparent diffusion coefficient (ADC) did not consider either the non-Gaussian water diffusion or the random flow of blood in capillaries, intravoxel incoherent motion (IVIM) and non-Gaussian MRI takes into account IVIM effects (low b values) and non-Gaussian diffusion effects (high b values) to explore the potential of perfusion MRI and non-Gaussian distribution. Lima et al. [9] first investigated the IVIM and non-Gaussian MRI in breast tissue. They reported that combining the two diffusion models as integrated biomarkers can improve the diagnostic value for the differentiation between malignant and benign breast lesions without the need for contrast medium and may also help understand the tumour biology.

Moreover, there has been a growing interest in texture analysis, which can be used to evaluate tumour heterogeneity by measuring the pixel grey-level on medical images [10]. Several studies have investigated the use of texture parameters from dynamic contrast-enhanced (DCE) images in breast cancer [11–13]. In addition, the standard ADC histogram analysis for the identification of malignant lesions and its relationship to molecular prognostic factors were evaluated in recent studies [14–16]. However, to our knowledge, investigators have not determined whether histogram analysis of both IVIM and non-Gaussian diffusion could identify HER2-positive breast cancer from other subtypes.

Thus, we used volumetric-tumour histogram-based analysis through IVIM and non-Gaussian diffusion MRI in breast cancer. The aim was to evaluate the imaging biomarkers of HER2-positive breast cancer in comparison to other molecular subtypes and further to determine the feasibility of histogram analysis to identify HR status in HER2-positive breast cancer.

## Materials and methods

### Patient population

This prospective study was received approval from the Institutional Review Board (1802181-7). Between January and November 2018, 154 patients, who were diagnosed with breast cancer or suspected of having breast cancer underwent breast MRI in preparation to receive NAC. No biopsy or previous neoadjuvant treatment was performed before the baseline MRI. The diagnosis of breast lesions was confirmed by core needle biopsy and the diagnosis of a suspicious lymph node was confirmed by ultrasound-guided fine needle aspiration. The exclusion criteria included the following: patients with no obvious lesion detected on MRI (n = 1), with signal quality that was too poor to process DWI (n = 2), with pathology revealing lymphoma (n = 1), without pathology and loss to follow-up (n = 5). Among all the included patients, three were confirmed to have bilateral breast cancer. For multicentric or multifocal tumours, the tumours with the largest sizes according to MRI were analysed. Finally, 145 patients with 148 lesions were enrolled in this study.

### Acquisition of DW images

All MRI was performed on a MAGNETOM Skyra 3 T MR system (Siemens Healthineers, Erlangen, Germany) using a dedicated 16-channel phased-array breast coil. The DWI in axial view was executed before contrast-agent injection using trace-weighted diffusion images (single-shot echo planar imaging) with spectral adiabatic inversion recovery for fat suppression were performed with the following parameters: b values (0, 10, 20, 50, 100, 150, 200, 400, 500, 800, 1000, 1500, 2000 s/mm^2^); repetition time/echo time, 5600/75 ms; flip angle, 90 degrees; field of view, 180 * 300 mm^2^; matrix, 96 * 200 mm^2^; slice thickness, 5.0 mm; 25 slices without gap; bandwidth, 1666 Hz; acquisition time, 6 min 21 s; generalized auto calibrating partially parallel acquisitions with an acceleration factor of 3; EPI factor, 96. The other sequences of breast MRI included a T1-weighted 2D gradient-echo and a fat-suppressed T2-weighted 2D fast spin-echo, as well as a fat-suppressed T1-weighted 3D fast spoiled gradient-echo sequence before and five times continuously after the contrast agent injection in the transverse plane, but these data were not considered for this study.

### Postprocessing of IVIM and non-Gaussian diffusion data

DWI data were inline calculated by the scanner integrated Syngo software (Siemens Healthineers) according to the monoexponential, biexponential and kurtosis models. The monoexponential diffusion model was calculated by the following equation: S_b_ = S_0_ exp (−b* ADC) where ADC represents the apparent diffusion coefficient, and S_0_ and S_b_ are the signal intensity values in the voxels with b values of 0 and 1000 s/mm^2^, respectively. The bi-exponential model was expressed by the following equation: S_b_/S_0_ = f exp(−b D*) + (1−f) exp(−b Dt), where Dt was the true diffusion, f was the perfusion fraction related to microcirculation and D* was the pseudo-diffusion coefficient which represents perfusion-related diffusion or incoherent microcirculation. The b-values used in IVIM are generally below 1000 s/mm^2^ (0, 10, 20, 50, 100, 150, 200, 400, 500 and 800 s/mm^2^). The non-Gaussian diffusion model was calculated by the following equation: S_b_ = S_0_ exp(−bMD + b^2^MD^2^ MKapp/6), where MD is the mean diffusivity and MK is the dimensionless metric mean kurtosis expressing the deviation from the Gaussian distribution. The b-values used in IVIM are generally high b values (0, 500, 1000, 1500 and 2000 s/mm^2^). Parametric maps, including ADC_1000_, Dt, f, D*, MK and MD maps, derived from the three diffusion models were generated with least squares fitting of all b-value data on a voxel-by-voxel basis with software.

### Volumetric-tumour histogram-based analysis in IVIM and non-Gaussian diffusion parameters

**Fig. 1:**
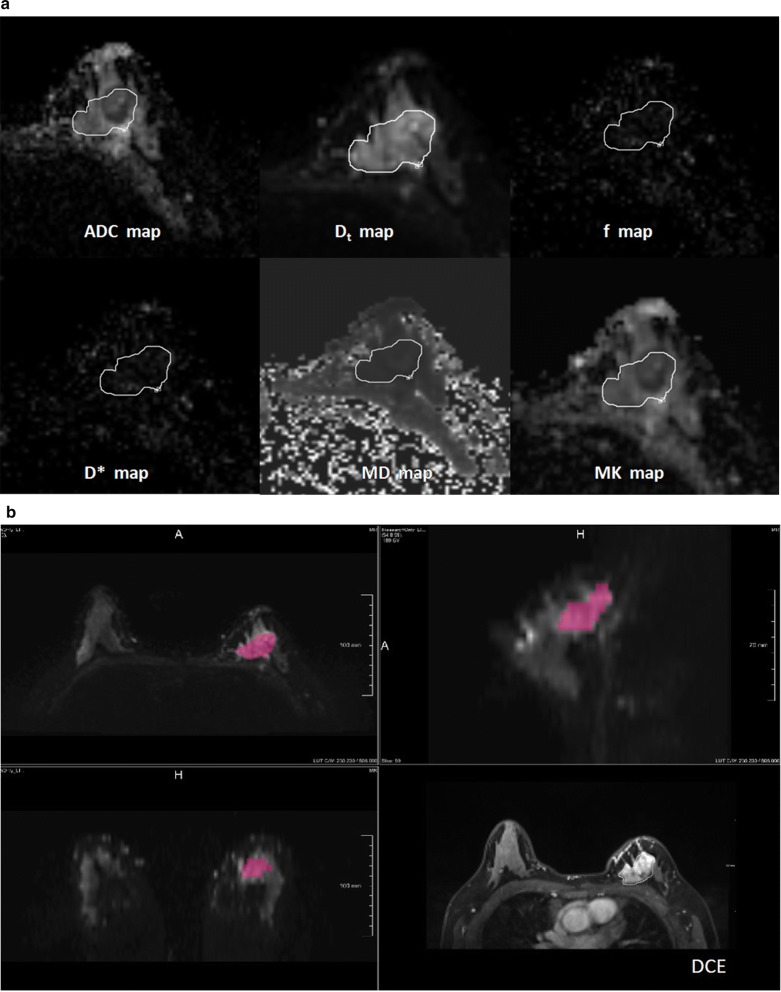
Workflow for the histogram analysis. **a** Foreground seed points were manually drawn on the axial view of the original ADC map (b value = 1000 s/mm^2^), and on the multiparametric diffusion parameter maps within the same ROI. **b** The 3D segmentation was created on the three multiplane reconstruction planes, and DCE images were accessed to verify the lesion boundaries

Histogram analysis was performed with the prototype MR Multiparameter Analysis software (Siemens Healthineers). The analysis of DWI-derived IVIM and non-Gaussian diffusion parameter maps were executed separately. Regions of interest (ROIs) were placed manually on the DW images with a b value of 1000 s/mm^2^, but DCE images to assist in locating the lesions and verifying the lesion boundaries. ROIs were placed on all slices that contained the whole tumour and the largest lesion (in the case of multicentric or multifocal tumours), and care was taken to avoid regions influenced by partial volume effect (Fig. 1). Two radiologists (C.Y. and Y.Q.C. with 6 and 2 years of experience in breast MRI, respectively) were blinded to the pathological and biochemical findings of each patient, and reviewed the MR images and draw the ROIs independently. When discrepancy of ROIs arose especially for non-mass enhancement lesion, two of them together made a consensus of lesion and redraw the ROIs later. The mean ROI of lesion for radiologist 1 was 37.72 ± 77.10, and the mean ROI of lesion for radiologist 2 was 45.24 ± 84.64. Spearman correlation showed ROI of tumour on DW image had good agreement with two radiologists (r = 0.835, p < 0.001). Finally, the data from the two radiologists’ average measurements were analysed.

Histogram analysis for the whole tumour on the parametric maps was performed and the parameters were extracted, including percentiles (5th, 50th and 95th of the ADC value), skewness (a measure of asymmetry of the probability distribution), kurtosis (a measure of the shape of the probability distribution), contrast (a measure of the signal difference) and entropy (a measure of texture irregularity).

### Histopathological analysis

All pathological results were defined according to the World Health Organization classification of breast tumor [17]. According to IHC-determined steroid HRs with estrogen receptor (ER), progesterone receptor (PR), and HER2 status, as well as tumour proliferation measured by Ki 67, breast cancer is considered to consist of four molecular types: (1) luminal A-like subtype (ER or PR positive, or both, HER2 negative, low proliferation); (2) luminal B-like subtype (ER or PR positive, or both, HER2 negative, high proliferation); (3) HER2 subtype, non-luminal (HER2 positive and ER and PR negative) or luminal (HER2 positive and ER or PR positive, or both); (4) basal-like subtype (HER2 negative and ER and PR negative; i.e. triple-negative breast cancer) [18].

### Statistical analysis

All data were analysed using SPSS 20.0 (Chicago, IL). Values of p < 0.05 were considered statistically significant. Categorical data were compared with the Pearson Chi squared test. IVIM and non-Gaussian diffusion parameters in the status of molecular prognostic biomarkers were compared by Student’s *t* test when normally distributed or by the Mann–Whitney *U* test when not normally distributed. Receiver operating characteristic (ROC) curve analysis was used to evaluate the effectiveness of IVIM and non-Gaussian diffusion parameters for differentiating HER2 positive breast cancer. The area under the curve (AUC), sensitivity, and specificity at the best cut-off point were reported. The Spearman correlation coefficient was calculated to analyse the correlations between of clinical TNM stage, and Ki 67 status with IVIM and non-Gaussian diffusion parameters.

## Results

### Patient characteristics

Of the 148 breast cancer lesions in 145 patients, 19 lesions (12. 8%) were triple-negative subtype, 14 (9. 5%) were luminal A subtype, 41 (27. 7%) were luminal B and HER2-negative subtype, 34 (23%) were luminal HER2-positive subtype, and 40 (27%) were non-luminal HER2-positive subtype. The clinical characteristics of the patients are listed in Table 1.

**Table 1:** Clinicopathological characteristics between the HER2- positive and HER2-negative groups

Variable	Overall (n = 148)	HER2(−) (n = 74)	HER2(+) (n = 74)	p value
Age (year)	49.68 ± 10.64	50.47 ± 11.15	49.04 ± 10.11	0.414
Diameter (cm)	3.42 ± 1.37	3.35 ± 1.20	3.48 ± 1.52	0.736
Affected side				
Right	64 (43.2)	30 (40.5)	34 (45.9)	0.507
Left	84 (56.4)	44 (59.5)	40 (54.1)	
T stage				
1	9 (6.0)	5 (6.7)	4 (5.4)	0.235
2	55 (37.2)	26 (35.1)	29 (39.2)	
3	30 (20.3)	11 (14.9)	19 (25.7)	
4	54 (36.5)	32 (43.2)	22 (29.7)	
N stage				
0	20 (13.5)	11 (14.9)	9 (12.1)	0.967
1	64 (43.2)	31 (41.9)	33 (44.6)	
2	26 (17.6)	13 (17.5)	13 (17.6)	
3	38 (25.7)	19 (25.7)	19 (25.7)	
M stage				
0	136 (91.9)	68 (91.9)	68 (91.9)	1.00
1	12 (8.1)	6 (8.1)	6 (8.1)	
cTNM				
1	4 (2.7)	2 (2.7)	2 (2.7)	0.685
2	44 (29.7)	25 (33.8)	19 (25.7)	
3	79 (53.4)	36 (48.6)	43 (58.1)	
4	21 (14.2)	11 (14.9)	10 (13.5)	
Ki 67				
	0.37 ± 0.20	0.35 ± 0.23	0.38 ± 0.18	0.360

Numerical data are presented as the mean ± SD. Nonnumerical data are presented as the number of patients (percentage). The p value was analysed between HER2-negative group and the HER2-positive group

*HER2* human epidermal growth factor receptor 2, *cTNM* clinical TNM stage

### Histogram analysis to differentiate HER2-positive breast cancer

**Fig. 2:**
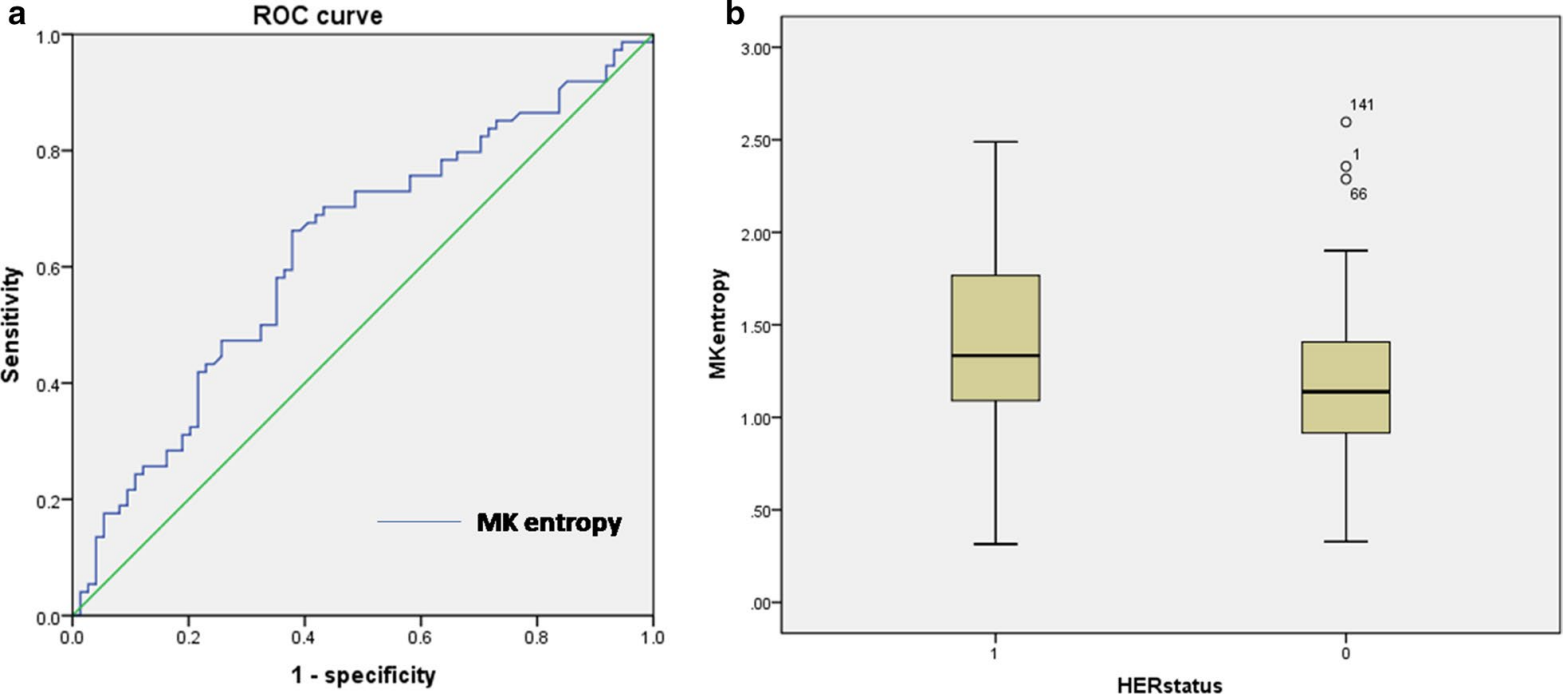
**a** Boxplots illustrating a comparison of MKentropy indexes between the HER2-positive and HER2-negative groups. **b** Receiver operating characteristic (ROC) curve for the differentiation of HER2-positive and HER2-negative groups using MK entropy for the entire tumour volume

There were 74 HER2-positive lesions and 74 HER2-negative lesions. Of all the histogram analyses of the IVIM and non-Gaussian diffusion parameters, only MK entropy was significantly higher in the HER2-positive group (1.39 ± 0.47) than in HER2-negative group (1.20 ± 0.45, p = 0.015) (Fig. 2a). The HER2 subtype differentiation yielded an AUC of 0.629 (95% CI 0.546, 0.707), a sensitivity of 62.6%, and a specificity of 66.2% (Fig. 2b). The other histogram parameters did not show a significant difference between HER2-positive and HER2-negative groups, which are detailed in the Additional file 1: table.

### Histogram analysis to differentiate HR status and lymph node metastasis status in HER2-positive breast cancer

**Fig. 3:**
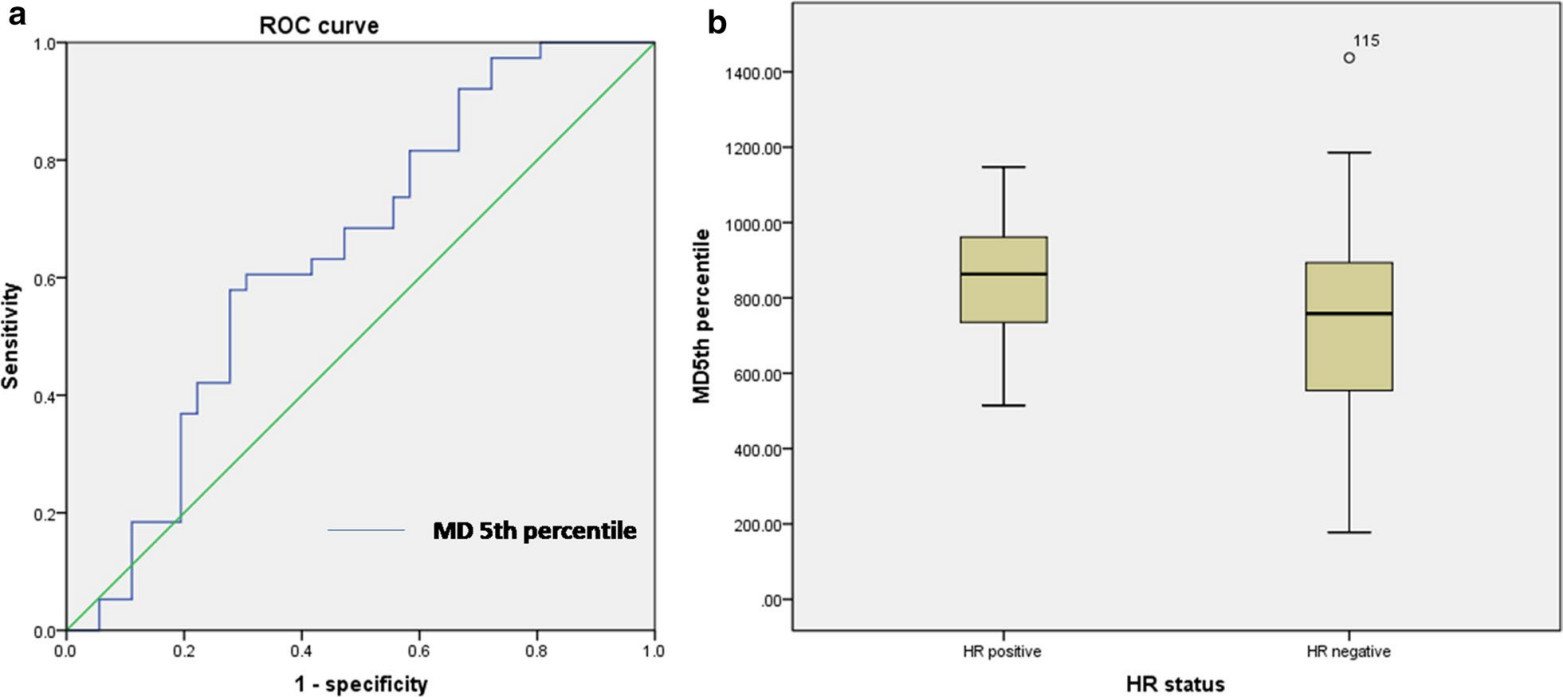
**a** Boxplots illustrating a comparison of MD 5th percentile indexes between HR-positive and HR-negative groups within the HER2 positive breast cancer patients. **b** Receiver operating characteristic (ROC) curve for differentiating HR status using MD 5th percentile for the entire tumour volume

For HR status, there were 34 HR-positive HER2-positive lesions and 40 HR-negative HER2-positive lesions. The MD 5th percentile was higher in the HR-positive group (850.12 ± 129.74) than in the HR-negative group within the HER2-positive breast cancer patients (804.12 ± 144.58, p = 0.041) (Fig. 3a). The HR differentiation yielded an AUC of 0.643 (95% CI 0.523, 0.751), a sensitivity of 72.22%, and a specificity of 57.89% (Fig. 3b).

**Fig. 4:**
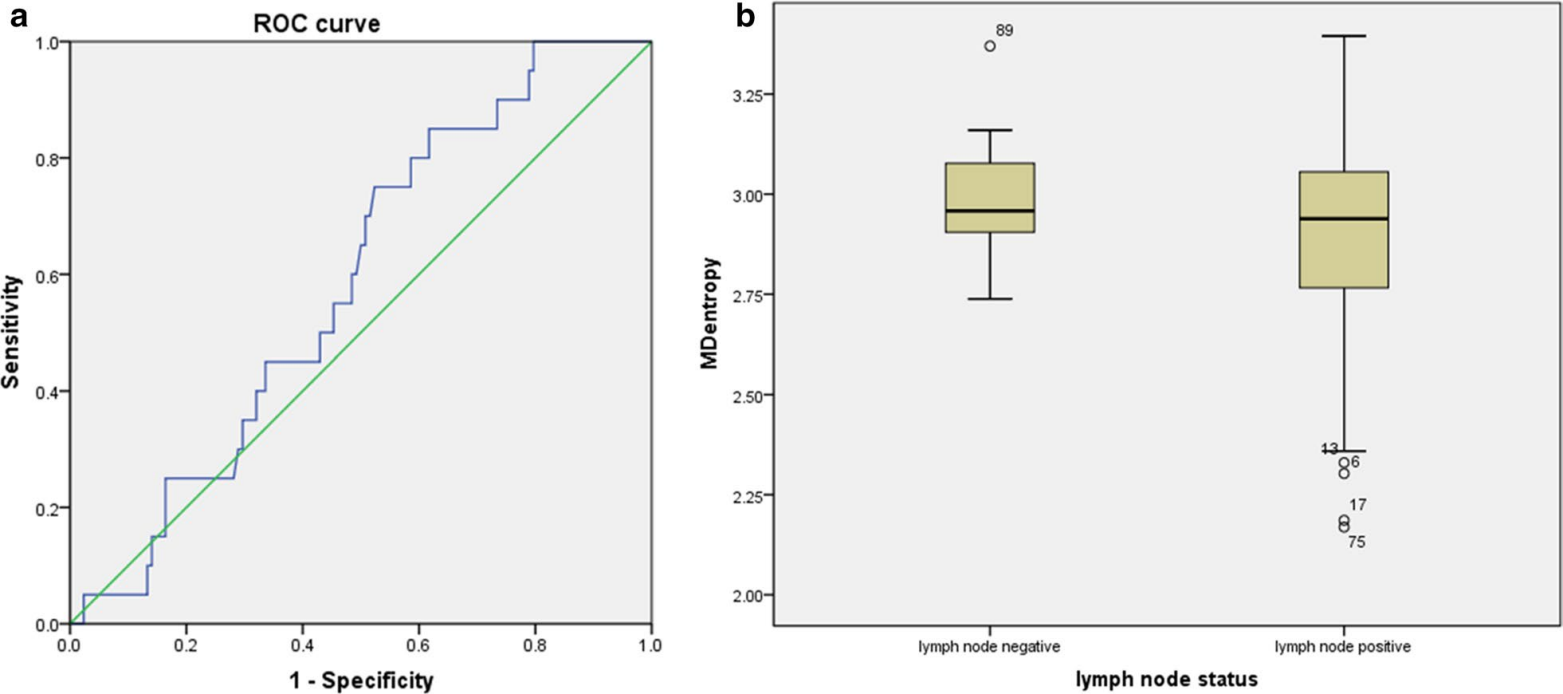
**a** Boxplots illustrating a comparison of MD entropy indexes between the lymph node-positive and -negative groups within HER2 positive breast cancer patients. **b** Receiver operating characteristic (ROC) curve for differentiating lymph node status using MD entropy for the entire tumour volume

**Fig. 5:**
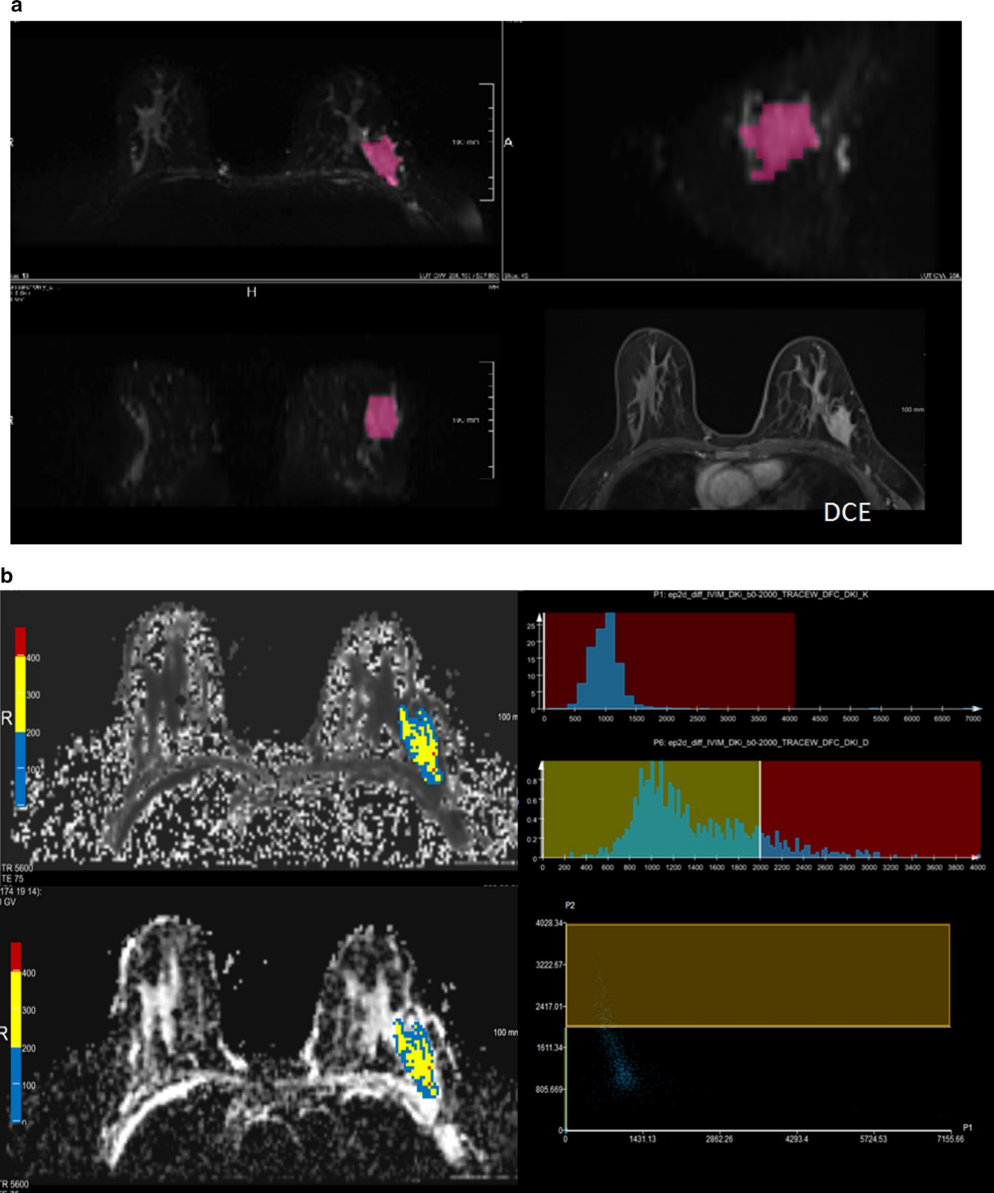
A 48-year-old female with human epidermal growth factor receptor 2 (HER2)-positive breast cancer, with hormone receptor (HR) -negative status and lymph node -positive status. **a** The 3D segmentation of the lesion was created on the three multiplane reconstruction planes, and DCE images were accessed to verify the lesion boundaries. **b** MK and MD maps overlaid with colour maps of ADC values and the histogram of whole-tumour MK and MD maps. The MK entropy was 1.83, the MD 5th percentile was 776.64 and the MD entropy was 2.13

For lymph node status, the MD entropy was lower in the lymph node positive group (2.20 ± 0.25) than in the lymph node-negative group (2.98 ± 0.15, p = 0.040) (Fig. 4a). Lymph node differentiation yielded an AUC of 0.587 (95% CI 0.504, 0.668), a sensitivity of 37.5%, and a specificity of 85% (Fig. 4b). The other IVIM and non-Gaussian diffusion parameters showed no significant difference in the differentiation of HR status or lymph node status in HER2-positive breast cancer, which are also detailed in the Additional file 1: table. The performances based on subtype and lymph node differentiation are shown in Fig. 5.

### IVIM and non-Gaussian diffusion parameters correlation with clinical TNM stage and Ki 67 index

The clinical TNM stage positively correlated with ADC skewness with an r of 0.28 (p = 0.000) and ADC kurtosis with an r of 0.21 (p = 0.006). Statistically, Ki 67 was negatively correlated with the 50th percentile and 95th percentile of ADC, 95th percentile of Dt, and the skewness and kurtosis of f, with r values of − 0.23 (p = 0.005), − 0.24 (p = 0.004), − 0.21 (p = 0.019), − 0.20 (p = 0.020) and − 0.25 (p = 0.004), respectively. Ki 67 was positively correlated with kurtosis of ADC, 50th percentile, 95th percentile and contrast of f, and with 5th percentile of MK, with r values of 0.19 (p = 0.020), 0.22 (p = 0.017), 0.20 (p = 0.013), 0.23 (p = 0.020) and 0.18 (p = 0.025), respectively (Table 2).

**Table 2:** Spearman’s correlation coefficients (r) of clinical TNM stage, Ki 67 and with combined IVIM and non-Gaussian diffusion parameters

	ADC		Dt		f		D*		MK		MD	
	cTNM	Ki67	cTNM	Ki67	cTNM	Ki67	cTNM	Ki67	cTNM	Ki67	cTNM	Ki67
50th	− 0.61	− 0.23*	− 0.04	− 0.11	0.04	0.22*	− 0.14	− 0.06	0.02	0.14	− 0.05	− 0.60
5th	− 0.23	− 0.14	− 0.02	− 0.08	0.04	0.13	− 0.04	0.01	0.05	0.18*	− 0.02	− 0.13
95th	− 0.40	− 0.24*	− 0.07	− 0.21*	0.04	0.20*	− 0.12	− 0.01	0.03	0.07	− 0.10	− 0.05
Skewness	0.28*	0.11	0.12	0.16	− 0.02	− 0.20*	0.05	− 0.01	− 0.12	− 0.07	0.16	− 0.04
Kurtosis	0.21*	0.19*	0.04	0.09	− 0.01	− 0.25*	0.09	− 0.02	− 0.12	− 0.13	0.06	− 0.01
Contrast	− 0.13	− 0.12	− 0.12	− 0.03	− 0.02	0.23*	− 0.10	− 0.02	0.01	0.12	− 0.01	0.12
Entropy	− 0.14	− 0.13	− 0.01	− 0.04	0.113	0.06	− 0.02	0.21	0.024	0.11	0.05	− 0.12

*ADC* apparent diffusion coefficient, *Dt* true diffusion coefficient, *f* perfusion fraction diffusion, D* pseudo-diffusion coefficient, *MK* mean kurtosis, *MD* mean diffusivity

*p < 0.05 was considered statistically significant

## Discussion

Our study found that histogram parameters of MD and MK from non-Gaussian diffusion maps can be used as potential biomarkers for differentiating HER2-positive subtypes and further for identifying the HR status and lymph node metastasis status in HER2-positive breast cancers. In the present study, the volumetric-tumour histogram parameters were also performed to assess the correlations with clinical TNM stage and Ki 67 in HER2-positive subtypes, such as the 50th, 95th percentile, skewness and kurtosis of ADC and f values, the 95th percentile of Dt value, the 5th percentile of MK value.

Technically, this study is the first to investigate the performance of volumetric-tumour histogram-based analysis on parameters derived from combined IVIM and non-Gaussian diffusion models. Due to the simplicity and ubiquity of the single component in DWI model, there has been increased use of advanced DWI techniques to assess the applicability to cancer diagnosis [8, 19–21]. Lima et al. first proposed the approach of combined IVIM and non-Gaussian diffusion MRI to better differentiate benign and malignant breast tumours; however, they did not further assess this combined advanced DWI model by histogram analysis [6, 9, 22–24]. Some other studies have investigated the heterogeneity of breast cancer using histogram analysis, which can potentially provide additional information beyond the mean values of ADCs, such as the skewness and kurtosis of the parameter distributions [25, 26]. Thus, the method of our study was based on the histogram-based analysis of the combined IVIM and non-Gaussian diffusion.

Our study was designed to mainly investigate HER2-positive breast cancer. Most previous DWI studies have mainly focused on ways to improve cancer detection and diagnosis, because malignant lesions are more cellular and vascular than benign entities [7, 8, 27]. Additionally, a few studies have focused on the relationship of DWI parameters with prognostic factors in breast cancer [28, 29]. A key finding in these reports is a significantly lower ADC value and lower ADC percentiles value in HR-positive tumours, which is speculated to be related to a lower perfusion contribution. However, no significant correlation was observed between HER2 status and the mean value of IVIM and non-Gaussian diffusion parameters. To date, a lack of evidences or studies further using a histogram analysis of advanced DWI models were applied to differentiate HER2 status especially, noting that understanding the heterogeneity of HER2-positive breast cancer can better guide targeted treatment. In our study, all the average values of ADC, IVIM and non-Gaussian diffusion showed no significant difference in HER2 status differentiation, which was in line with the findings of Lima et al. [23]. Partially different from the findings of Kim et al. and Martincich et al., they reported that higher ADC value were common seen in HER2-positive tumours due to the effect of higher tumour blood flow and an increased volume of extracellular fluid [14, 30]. Furthermore, the entropy of MK from histogram parameters was significantly higher in the HER2-positive group than in the HER2-negative group. The possible explanation was that the higher entropy of MK, represented the increased heterogeneity, was associated with increased angiogenesis and necrosis through induction of vascular endothelial growth factor (VEGF) caused by HER2 expression [31, 32]. Therefore, our findings revealed that the entropy of MK derived from histogram analysis can identify HER2-positive subtypes, which were essential for guiding the targeted therapy of trastuzumab.

Owing to the new results of the APHINITY study, trastuzumab combined with pertuzumab has been shown to be more effective than trastuzumab alone, especially in patients with HR-negative and lymph node-positive status [33]. Thus, to select suitable patients for dual-target treatment among HER2-positive breast cancer patients, we further investigated the feasibility of histogram analysis for differentiating the HR receptor and lymph node statuses. Our study found that the 5th percentile of MD was lower in the HR-negative group and that the entropy of MD was lower in the lymph node-positive group. For HER2-positive patients, the status of HR-negative and lymph node-positive may result in a more aggressive tumours and higher risk for recurrence and metastasis [34]. The lower value of the 5th percentile and entropy derived from the MD of the non-Gaussian diffusion model represents a decrease in the overall water molecule diffusion and an increase in the diffusion resistance. Kim et al. [35] also found that lower tumour ADC values were associated with the presence of lymph node metastasis in invasive ductal carcinoma, which was partially in line with our findings because MD was corrected by non-Gaussian distribution. Lower histogram indexes from ADC value were related to increased microenvironmental stiffness that may reflect a restriction of water diffusion in a breast tumour.

Furthermore, our study investigated the relationship between histogram parameters and clinical TNM stage, as well as the Ki 67 index. Our results showed a positive correlation between the clinical TNM stage and tumour skewness and kurtosis of ADC values, which were consistent with previous observations [27]. High-grade tumours are characterized by the absence of tubule and gland formation, marked variation of nuclear pleomorphism, and high mitotic counts. These changes represent the increasing tissue complexity at the microstructural level, thereby manifesting higher kurtosis and skewness of ADC in tumour. Additionally, we further reported the correlation between multiparametric DWI parameters and Ki 67 index. Partially consisted with previous observations, a high Ki 67 index was related to significantly lowered 50th percentile and 95th percentile of ADC values, 95th percentile of Dt value and to skewness and kurtosis of f value [14, 29]. Meanwhile, our study also found several other histogram parameters that are positively correlated with the Ki 67 index, such as kurtosis of ADC, 50th percentile, 95th percentile and contrast of f, and with 5th percentile of MK. As we known, the Ki-67 index is correlated with a high mitotic count and recurrent disease and can be considered as a marker evaluating the degree of cellularity. Some membrane activity between cells might contribute to DWI parameter behavior. These findings still need further studies to better understand the special clinical significance of these histogram parameters.

There are several limitations in this study. First, for multicentric or multifocal tumours, small lesions with a diameter less than 5 mm were not included due to the slice thickness and limited resolution of DWI. Readout-segmented DWI is required to assess smaller lesions in further studies. Second, only the first-order histogram-based features of DWI were extracted in this study. Even though a few features were found to be significant, the AUC values were not high enough. More extensive observations of these relationships are needed to confirm these findings according to increase the number of samples. The histogram-based features of DCE images and T2WI, as well as the higher-order texture features, are still warranted. Meanwhile, based on the current findings of these statistical indexes, the future study hopes to expand the sample size in order to make the trend more obvious and improve the diagnostic efficiency. Third, most of the patients in this study received NAC, and follow-up MRI examinations were still being performed. This study focused on the histogram-based features of HER2-positive breast cancer. The diagnostic value of histogram features to predict the response to NAC is needed in the future.

## Conclusion

In conclusion, volumetric-lesion histogram analysis of IVIM and non-Gaussian diffusion, especially for the non-Gaussian diffusion model, may be useful in providing prognostic information about HER2-positive breast cancers, thus potentially contributing to individualized anti-HER2 targeted therapy plans and might play an important role in evaluating the NAC response. Further studies still are needed.

## Additional file

**Additional file 1.** All the parameters of histogram analysis for the whole tumour in different groups.

## Data Availability

The datasets generated during the current study are not publicly available due to the patient confidentiality but are available from the corresponding author.
